# Design Preferences, Routines, and Well-Being of Older Adults Using Voice-Guided Digital Mindfulness: Qualitative Interview Study

**DOI:** 10.2196/67533

**Published:** 2025-06-03

**Authors:** Lucy McCarren, Sanna Kuoppamäki

**Affiliations:** 1 Department of Biomedical Engineering and Health Systems School of Engineering Sciences in Chemistry, Biotechnology and Health KTH Royal Institute of Technology Huddinge Sweden

**Keywords:** mindfulness-based interventions, mobile health, mHealth, digital mindfulness, mindfulness app, older adults, qualitative study

## Abstract

**Background:**

Mindfulness-based interventions have been demonstrated to be effective in improving bodily and emotional well-being. However, only a few studies have explored individual differences in the application and use of digital mindfulness among adults aged ≥65 years. Voice-guided mindfulness technologies can increase the accessibility of mindfulness training, but the expected benefits may not be similar in all user groups.

**Objective:**

This study aims to understand how older adults incorporate mindfulness into their habits and routines, explore the digital mindfulness design preferences of older adults, and understand if and how digital mindfulness can facilitate self-perceived well-being among older adults.

**Methods:**

A qualitative interview study built on an interpretive-constructivist paradigm was conducted among older adults in Sweden who used a voice-guided mindfulness app for a 3-week period in their homes (N=15). Semistructured interviews were conducted one-on-one with participants after using the app. Qualitative thematic analysis was used to explore the lived experiences of digital mindfulness, as articulated by participants, which allowed an open exploration of the subjective experiences of digital mindfulness and their possible effects and outcomes.

**Results:**

From the coding stage, 23 codes describing the digital mindfulness experience were identified from the data. These codes were thematized to group the codes together, resulting in 7 subthemes. From these 7 subthemes, three main themes were formed to answer the research objectives: (1) the embeddedness of digital mindfulness in older adults’ daily routines and habits, (2) heterogeneity in older adults’ design preferences for digital mindfulness, and (3) the consequences of digital mindfulness on the self-perceived well-being of older adults.

**Conclusions:**

This study concludes that digital mindfulness offers a possibility to enhance the self-perceived well-being of older adults by fostering resilience and self-care. However, adverse effects of mindfulness, such as frustration and discomfort, can also be experienced by older adults. The digital mindfulness experiences and preferences of older adults are highly individual, diverse, and manifold, which indicates that personalized approaches are essential for effective engagement. By acknowledging and addressing the heterogeneous design preferences within this demographic, developers can create more personalized and adaptive voice-guided mindfulness apps.

## Introduction

### Overview

Mindfulness, which can be broadly defined as a state of focusing one’s awareness on the present moment [1,2], has gained increasing attention in the field of digital health and mental well-being. Practicing mindfulness has been shown to be associated with reduced levels of stress, anxiety, and depressive symptoms across different population groups, demonstrating the benefits of mindfulness for mental well-being [3-6]. Consequently, digital mindfulness technologies, such as mindfulness apps, virtual reality environments, and wearables, have emerged, which enhance the mindfulness experience and improve the accessibility of mindfulness practice [7,8].

Mindfulness technologies can be considered as digital self-care technologies through which users can engage in the maintenance of physical and mental well-being and prevention of chronic diseases without needing human contact with a health care provider [9]. Self-care is defined as the promotion and maintenance of health and the prevention of disease, with or without the support of health care providers [10,11]. Self-care technologies such as mobile apps can provide a medium for users to collect, monitor, and track health-related behaviors independently, along with guidance and advice on areas that lack accessibility to care professionals or therapy [12,13]. The uptake of digital self-care technologies nevertheless depends on an individual’s digital literacy; access to technology; and social, cognitive, and attentional resources, which are unevenly distributed across different social groups [14,15].

Older adults, defined as a group of adults aged ≥65 years, are recognized as a group who could benefit from digital mindfulness. Previous research has mostly explored mindfulness-based interventions (MBIs) from the medical or clinical perspective, demonstrating the clinical effects of mindfulness training for older adults [16-18], whereas only a few studies have explored the use of a mindfulness app with older adults using a qualitative approach [19]. Despite the increasing amount and variance of digital health apps in the market, mindfulness apps are not equally used by all groups. Factors such as age, education, gender, eHealth literacy skills, and privacy concerns may play a role in mobile health (mHealth) app use [20]. The accessibility of digital mindfulness for older adults remains a challenge, as the benefits are influenced by factors such as digital skills and interest toward technology [18]. As a result, the age-specific characteristics of using digital mindfulness have largely remained unexplored, particularly from the perspective of self-perceived benefits and challenges of digital mindfulness.

To fill the aforementioned research gap, we conducted a qualitative interview study to explore voice-guided digital mindfulness among older adults in Sweden. The main objectives are as follows: (1) understand how older adults incorporate mindfulness into their habits and routines, (2) explore the digital mindfulness design preferences of older adults, and (3) understand how digital mindfulness can facilitate self-perceived well-being among older adults. By considering the concept of aged heterogeneity [21,22], our research addresses the individual differences in the experiences and preferences of older adults when using a voice-guided mindfulness app over a 3-week period in their homes.

### Background

#### MBIs in Medical and Clinical Research

Mindfulness originates from the Buddhist practice of meditation [23]. Kabat-Zinn [1], an early pioneer of modern mindfulness in the West, developed mindfulness-based stress reduction (MBSR) in 1979. Kabat-Zinn [1] defines mindfulness as “paying attention in a particular way, on purpose, in the present moment, and nonjudgmentally.” In clinical psychology, Bishop et al [2] proposed a 2-component model of mindfulness in order to produce an operational definition. The first component involves the focus of attention so that it is maintained in the present, and the second component involves an accepting and open attitude toward the experiences in the present moment. The Swedish Healthcare Guide (1177 Vårdguiden) describes mindfulness as a method that involves focusing on the present with the help of senses and breathing, which can help an individual develop the ability of conscious presence.

In medical and clinical research, MBIs describe the use of mindfulness training in order to promote health and treat and prevent health-related conditions [6]. MBIs in contemporary psychology have been adopted as a form of mental training to reduce cognitive vulnerability to reactive modes that might otherwise heighten stress and emotional distress [2]. Consequently, MBIs have been developed in the form of mindfulness-based programs such as MBSR and mindfulness-based cognitive therapy for the prevention and treatment of mental health conditions [24].

Mindfulness practice can be considered a holistic way to support the well-being and resilience of older adults [25]. Research shows that MBIs are effective as a treatment for physical symptoms that often occur in later life. In Sweden, Norman et al [26] showed that mindfulness training decreased symptoms of fatigue, dizziness, and shortness of breath in a group of adults aged >70 years with chronic heart failure. Henriksson et al [27] explored the association between mindfulness training and the reduction of chronic pain among individuals aged ≥50 years. The group assigned to mindfulness training showed increased mindfulness skills, reduced pain intensity, reduced pain-related interference, heightened pain acceptance, reduced affective distress, and higher ratings of life satisfaction following the training.

MBIs can also improve mental and emotional well-being in later life. Studies have offered support for the use of MBIs to relieve anxiety, depression, and stress and improve pain acceptance [7,17,28,29]. Randomized controlled trials show that MBIs can improve positive affect and reduce symptoms of anxiety and depression for older adults [7,28]. MBIs can also enhance cognitive functioning [29] and working memory for older adults [8], alleviate chronic insomnia, and improve sleep quality [7,30]. Therefore, mindfulness, by definition, can be considered as an embodied experience, where bodily states shape emotion and cognitive processes [23]. For mindfulness research, this highlights the reciprocal relationship between the mind and body.

Medical and clinical research has demonstrated the positive effects of mindfulness on well-being, but it remains unclear whether these benefits are strengthened by regular practice and if harmful effects can occur [31]. Galante et al [31] have demonstrated that a wide range of experiences can arise with mindfulness practice, ranging from profoundly positive to challenging and potentially harmful experiences. Unpleasant experiences followed by mindfulness practice have been reported to a relatively high degree in nonclinical contexts. These negative impacts can vary from anxiety issues to traumatic re-experiencing and emotional sensitivity [32]. Among mindfulness app users, reported effects included reduced self-efficacy, lower mood, increased frustration, and inability to control thoughts caused by the lack of supervision associated with self-guided digital mindfulness [33].

Consequently, the expected benefits of mindfulness training are mediated by various individual, social, and cultural factors [31]. At the individual level, the benefits and experiences of mindfulness training are influenced by the duration of practice, previous mindfulness experience, and health conditions [5,17,32]. At the sociodemographic level, social class, education, and cultural background can influence the mindfulness outcomes; yet, these factors have been less studied in previous mindfulness research. For older adults, these mediators in the mindfulness experience can also include familial and social relationships and a sense of community [34]. For these reasons, it becomes important to explore digital mindfulness with a qualitative approach and recognize older adults’ experiences with digital mindfulness in relation to their background.

#### Digital Mindfulness for Older Adults

Digital mindfulness is a specific type of mindfulness training that includes the provision of MBIs through a digital platform, such as a mobile app. Mindfulness apps have increased the availability of MBIs to the public with and without mental health conditions. The most implemented techniques in mindfulness apps are voice-guided mindfulness exercises, self-tracking of mindfulness activities, setting personal goals, and receiving notifications [35]. These activities aim to track and influence the user’s emotional state through increasing reflexivity and emotional awareness. Mindfulness apps mostly consist of a self-guided format, where users do not have a personal interaction with a mindfulness instructor [36].

Digital MBIs can increase accessibility and early prevention of mental health, as the provision of digital MBIs makes mindfulness easily accessible and could constitute a feasible alternative to promote mental health at a large scale [37]. However, the success of digital MBIs is reliant on adequate user engagement with digital mindfulness technologies. Several studies have explored mindfulness app users in their everyday contexts, demonstrating the positive effects of the use of mindfulness apps on the overall well-being [38,39]. The continuity of the mindfulness app use nevertheless remains a challenge. It is estimated that <5% of individuals continue using the mindfulness app after 30 days of download [40].

Older adults may have distinct characteristics of engagement with mindfulness apps [16,18,19]. Aged heterogeneity in information and communication technology refers to differences in older adults’ backgrounds regarding the resources, needs, skills, and motivation to adopt digital technologies into their daily lives [22,41]. Older adults’ use and perceived benefits of mindfulness apps are influenced by their general perceptions and attitudes toward technology, self-efficacy, and skill level [16]. Older adults may use mindfulness apps to improve interpersonal relationships [18,34]. Voice as an interaction modality has been shown to decrease many technology adoption barriers among older adults [42]. However, the conversational style can significantly impact the perceived likability of voice-guided technologies for this age group [43].

## Methods

### Overview

The app used for the study is Mindfulnessresan, which is a commercially available mobile app available from the Apple App Store. The app was provided free of charge to participants for the duration of the study. Mindfulnessresan was developed by a behavioral scientist and certified mindfulness instructor from the University of Gothenburg. The app has 5- to 10-minute voice-guided mindfulness exercises in Swedish (42 total exercises), with a new exercise available to the user each day. The guided mindfulness practices are based on Vipassana practices [44] and are inspired by the MBSR course by Kabat-Zinn [1]. The mindfulness exercises had varying themes, including focusing on breath, mentally scanning the body, handling negative thoughts, and cultivating a nonjudgmental orientation toward thoughts and experiences.

This study was conducted with 15 participants who reported as volunteers to take part in a 3-stage study using a voice-guided mindfulness app. The sample size was determined based on the principles of purposive sampling, which indicates the selection of “information-rich” cases for in-depth analysis [45]. We considered that the sample size was large enough to allow a new understanding of the self-perceived experiences of the mindfulness app among this age group but small enough to conduct a more in-depth analysis of interview material [46]. In qualitative research, the number of 20 research participants is often considered the saturation point after which little or no new information is generated among participants in one analytically relevant category [45]. In our study, we recruited 20 older adults to take part in all 3 stages of the study, and 15 (75%) participants completed all stages of the study. We aimed to reach a saturation point through the interview procedure, emphasizing open-ended questions, which have shown to contribute to the richness of data.

The study procedure consisted of three stages: (1) preuse study, (2) the use of a voice-guided mindfulness app over a 3-week period, and (3) postuse study. In the preuse study, participants received helped to install the mobile app on their phones, and filled out a questionnaire investigating their previous use of mHealth apps and self-perceived socioemotional well-being. Perceived socioemotional well-being consists of measuring well-being (World Health Organization-Five Well-Being Index [WHO-5]) and mindfulness (Five Facet Mindfulness Questionnaire [FFMQ] scale). In addition, sociodemographic data variables were collected (age, gender, civil status, and household type).

In stage 2 of this study, participants used the mindfulness app daily for a 3-week period in their homes. Participants were given verbal and written instructions for using the app. Participants were instructed to engage with the mobile app daily at a time of their convenience, but their actual use was not monitored. In this procedure, we expected to better understand participants’ authentic use of the app instead of giving participants predefined time frames or strict adherence rules. Participants with Android phones (8/15, 53%) were unable to download the app, as it was available only on the Apple App Store. Hence, those participants accessed screen recordings of the app through a shared drive, where they could follow the same exercises via a file-sharing app on their Android phones.

After having used the mindfulness app for a 3-week period, participants took part in the postuse study by answering a questionnaire and a semistructured interview. The interviews were conducted one-on-one with each participant and lasted approximately 20 minutes. The interviews were audio recorded, and all audio recordings were transcribed for analysis. The data collected during the postuse study consisted of the same perceived socioemotional well-being questionnaires as collected in the preuse study. In addition, participants answered the mHealth App Usability Questionnaire [47].

### Analytical Approach

The study analyzed the research material through a qualitative approach, which aimed to address the complexities and subjective meanings of user engagement with a mindfulness app. Qualitative approaches have generated increasing interest in mindfulness research to explain the multifaceted characteristics and embodied nature of mindfulness through the interpretive-constructivist paradigm [48]. Qualitative approaches can provide alternative ways to explore the various subjective experiences of digital mindfulness to complement the medical model of MBIs [49]. They can be considered particularly valuable in addressing the heterogeneity in mindfulness practices among vulnerable populations, which often remain underrepresented in quantitative or medical research [50,51]. This study is reported in accordance with Standards for Reporting Qualitative Research guidelines [52].

The interpretive-constructivist paradigm in mindfulness research draws attention to subjective perceptions and experiences articulated with and through language [48]. The interpretive-constructivist paradigm indicates that the experience of digital mindfulness is socially constructed within language, and the well-being outcomes are subjectively perceived. Therefore, the narratives articulated by older adults can provide detailed accounts on how the subjective experiences of health and well-being are formed under certain contexts or environments [49] or mediated by social or cultural factors [53]. It can also mitigate the potential for a power imbalance between researchers and vulnerable groups as a research population [51] by giving research participants the opportunity to define and identify the experiences of digital mindfulness themselves.

### Participant Recruitment

Participants were recruited by sending an email invitation to older adults through one Stockholm municipality that organizes weekly meetings for older adults aged ≥65 years. The Stockholm municipality, through which we recruited participants, represents an average municipality in Stockholm in terms of the sociodemographic background of the population. In the email invitation, we invited older adults interested in mindfulness to join a workshop at their weekly meeting for older adults. Eligibility criteria required participants to be (1) aged ≥65 years, (2) fluent in Swedish, and (3) smartphone owners. We used convenience sampling, selecting participants on a first-come, first-served basis. In the workshop, we presented the project and the Mindfulnessresan app and invited volunteers to take part in the study. From this group, 15 older adults were enrolled in the study and signed an informed consent form. We helped them download the app to their phones and gave verbal and written instructions for using the app. In addition, 5 participants were recruited through distributing the invitation in private yoga studios in Stockholm, following the same procedure as those who were recruited through Stockholm municipality. By recruiting participants in 2 separate locations, we were able to reach a relatively diverse sample of older adults in terms of their sociodemographic background, as shown by Table 1. Of the 15 participants recruited through Stockholm municipality, 10 (67%) completed the study. All (5/5, 100%) participants recruited from yoga studios completed the study.

**Table 1:** Participants’ sociodemographic characteristics (age and gender), previous mindfulness experience, previous mobile health (mHealth) use, self-perceived well-being scores (World Health Organization-Five Well-Being Index [WHO-5]), and mindfulness scores (Five Facet Mindfulness Questionnaire [FFMQ]; N=15).

ID	Age (y)	Sex	Previous mindfulness experience	Previous mHealth use^a^	WHO-5 (0-100)	FFMQ score (0-100)
P1	65	Female	Yes	Intermediate	High (100)	73
P2	65	Female	Yes	High	High (90)	58
P3	66	Male	No	High	High (100)	58
P4	75	Male	Yes	Intermediate	Low (45)	45
P5	66	Female	Yes	Intermediate	High (90)	68
P6	71	Male	No	High	High (95)	60
P7	74	Female	Yes	Intermediate	Intermediate (65)	65
P8	83	Female	Yes	High	Low (40)	70
P9	69	Female	Yes	Intermediate	High (90)	73
P10	78	Female	No	Low	High (100)	58
P11	79	Female	No	Low	High (100)	70
P12	66	Female	No	High	High (95)	65
P13	89	Female	No	High	Intermediate (75)	50
P14	74	Female	Yes	Low	Intermediate (55)	63
P15	77	Female	Yes	Low	High (95)	67

^a^Previous mHealth use is calculated from participants’ previous use of mobile technology for health, where daily use indicates high use, 1 to 3 times per week indicates intermediate use, and <1 to 3 times per week indicates low use.

### Participant Characteristics

Participants’ sociodemographic details are reported in Table 1. Most (12/15, 80%) of the participants were female, and just over half (8/15, 53%) of the participants had previous experience with mindfulness. Most (9/15, 60%) of the participants lived alone, and only 6% (1/15) of the participants lived in a senior living facility. Only 40% (6/15) of the participants were married or in a domestic relationship. Most (10/15, 66%) of the participants evaluated their self-perceived well-being as high (WHO-5 score >75). In total, 20% (3/15) of the participants reported an intermediate well-being (WHO-5 score between 50 and 75). Only 15% (2/15) of the participants reported a low well-being (WHO-5 score ≤50).

In the pre- and postuse study, participants were asked to fill out a survey questionnaire consisting of questions on their previous use of mobile apps, perceived well-being (WHO-5 score), perceived mindfulness (FFMQ scale), and sociodemographic information.

The FFMQ scale is based on a factor analytical study of 5 independently developed mindfulness scales that represent different elements of the conceptualization of mindfulness [54]. The 5 factors value the respondent’s degree of observation, description, ability to act with awareness, nonreactivity, and nonjudgment toward inner thoughts and feelings. For this study, a shortened version of the FFMQ scale was used, containing 15 questions. The FFMQ-15 shows valid psychometric properties and can therefore be used in both health care settings and research settings [55]. The raw score was scaled to give a final score between 0 to 100.

The WHO-5 index uses five self-reported measures of overall mental well-being [56]: (1) “I have felt cheerful and in good spirits,” (2) “I have felt calm and relaxed,” (3) “I have felt active and vigorous,” (4) “I woke up feeling fresh and rested,” and (5) “My daily life has been filled with things that interest me.” There are 5 possible answers, including “daily” (score 4), “1-3 times per week” (score 3), “1-3 times per 2 weeks” (score 2), “more seldom” (score 1), and “never” (score 0). The scores for the 5 questions are summed to obtain a raw score for each participant, and the raw score, ranging from 0 to 20, is multiplied by 5 to give the final score, where 0 represents the lowest well-being and 100 represents the highest.

Results from the prestudy questionnaire revealed participants’ previous experience with mobile technology (smartphone, tablet, and portable device), mHealth apps, and conversational agents. Approximately half (6/15, 40%) of the participants used mobile technology daily for health reasons. Only a few participants used mobile technology daily for mental well-being (3/15, 20%), whereas 40% (6/15) of the participants had never used mobile technology for mental well-being. Participants rarely used mHealth apps, except for the Swedish health advice service app, which was used by six (6/15, 40%) of the participants 1 to 3 times per month. Only a few participants had used mHealth apps for tracking steps and sleep and practicing yoga (3/15, 20%). Seven of the participants (7/15, 46%) had used a voice assistant before. The voice assistants they had tried were Google Home, Siri, and ChatGPT. Of those participants who had never tried to use voice assistants, the most common reasons were that they did not need voice assistants or they did not know how to use them.

### Data

#### Semistructured Interviews

In the postuse study, participants were interviewed about the user experience of the mindfulness app and the self-perceived benefits of digital mindfulness training. A semistructured interview topic guide (Multimedia Appendix 1) was prepared, which was guided by previous mindfulness literature. We conducted the interview with an open-minded approach, including questions that would enable the informants to respond as they found suitable. The interview guide was structured around the following themes and questions: (1) perceived health, (2) routines, (3) design preferences and usability of Mindfulnessresan, (4) digital mindfulness and well-being, and (5) ethical considerations.

#### Thematic Analysis of Semistructured Interviews

The interviews were conducted, transcribed, and analyzed by 2 researchers using an investigator triangulation method. Investigator triangulation refers to the participation of ≥2 researchers in the same study to provide multiple observations and conclusions that can serve as a strategy to validate the trustworthiness of research findings [57]. Researchers conducting the analysis had complementary expertise in social science methodologies and computer science. One of the researchers had a previous background in serving as a mindfulness instructor.

A thematic analysis was used to detect codes and themes from the interview data through identifying, analyzing, and interpreting patterns [58]. Qualitative thematic analysis is an established method in health research to explore individuals’ beliefs, perspectives, and experiences within certain social and cultural contexts [59]. Analysis followed the principles of reflexive thematic analysis with 6 distinct stages described: familiarizing with the dataset; coding the dataset; generating initial themes; developing and reviewing themes; refining, defining, and naming themes; and writing up [59]. Data saturation was discussed throughout these stages, with the notification that no new codes or themes emerged from the data after stages 3 and 4 [45].

The interview transcripts were thematized inductively, indicating that codes were generated from the data to allow patterns and themes to emerge from the data without using any predetermined code lists or framework in the analysis [55]. After both researchers had familiarized themselves with the transcriptions, codes that answered the research objectives were identified from the data. This initial coding was conducted on the entire interview material, and certain keywords were selected to represent the codes (eg, “creating routines,” “voice,” and “sound”). To further recognize patterns in participants’ statements, initial themes were generated that gathered codes together in a way that was representative of the data and research questions (eg, “mindfulness as a therapeutic intervention” or “mindfulness coach persona”). In developing and reviewing the themes, researchers jointly discussed the refining and naming of the themes to involve a level of interpretation and conceptualization in the analysis. Thus, the final themes described the patterns in the participants’ experience of digital mindfulness that emerged from the data through interpretative analysis. The codes and themes are presented in Multimedia Appendix 2.

### Ethical Considerations

The research was approved by the Swedish Ethical Review Authority (reference number 2023-07682-01). All participants gave informed verbal and written consent for participation in the study and publication of anonymized extractions from the data. Before participants signed the informed consent, they were given both written and verbal information about the effects of practicing mindfulness and potential risks in participating in the study. Participants were given the opportunity to ask questions and withdraw from the study at any time. The privacy and confidentiality of participants were protected by giving all participants an anonymous ID that was used in the analysis and reporting of the results. Participants were given a small financial compensation of a gift card worth 200 SEK (US $20.82) after they completed their participation in the study. The amount of financial compensation was not specified in the recruitment material.

## Results

### Overview

**Figure 1:**
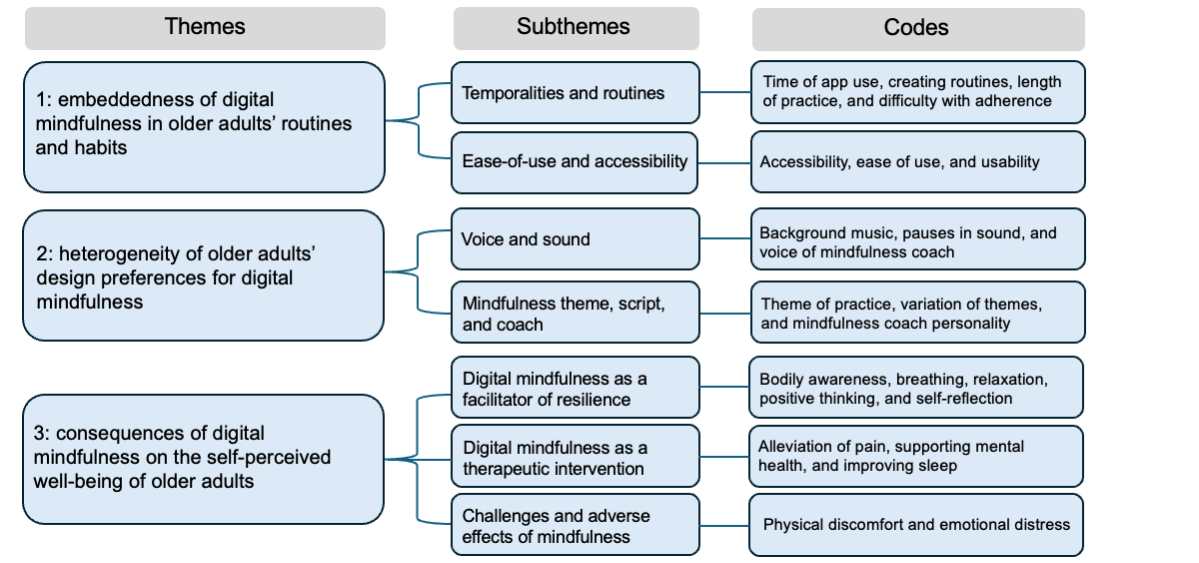
A map showing the themes, subthemes, and codes that emerged from the thematic analysis of the semistructured interview data.

From the coding stage, 23 codes describing the digital mindfulness experience were identified from the data. These codes were thematized to group the codes together, resulting in 7 subthemes. From these 7 subthemes, three main themes were formed to answer the research objective: (1) the embeddedness of digital mindfulness in older adults’ daily routines and habits, (2) heterogeneity in older adults’ design preferences for digital mindfulness, and (3) the consequences of digital mindfulness on the self-perceived well-being of older adults. Codes, subthemes, and main themes are presented in Figure 1 and Multimedia Appendix 2.

### Theme 1: Embeddedness of Digital Mindfulness in Older Adults’ Daily Routines and Habits

#### Temporalities and Routines

Participants described the use of the app as relatively effortless, because “It takes only 5 minutes so I have time to do it” (P4; male participant aged 75 y). They were able to choose the time of the day for training based on their own preferences. Participants described how the temporality of the app use could significantly influence the experience of mindfulness training. Two participants mentioned that practicing mindfulness in the evening instead of morning can impact the perceived effects of the training:

At first, I did it in the evening, but then I started doing it in the morning instead. I almost thought it felt better. Because in the evening, when you do it as the last thing you do, then you end up not hearing what he says, you almost fall asleep.

P3; male participant aged 66 y

If you listen during the day, you feel a little more calm during the day. But if you listen in the evening, you might sleep better.

P6; male participant aged 71 y

Participants recognized that continuity with the app use and embedding the app into the daily routines elicited a sense of well-being:

Doing it regularly and creating routines [with the app use] feels good.

P4; male participant 75 y

In addition, a participant described that “It worked quite well actually because I did it as a sort of morning routine. [...] I went back to bed and completed the exercise there. It was a very relaxing and pleasant start to the day” (P9; female participant aged 69 y).

The app provided participants with flexibility in finding a suitable time and location for practice, which seemed to improve the user experience. One participant described that practicing mindfulness in familiar locations, such as when lying in bed, helped in creating routines with the app, which contributed to improved user engagement. Mindfulness practice requires concentration, which is easier to do in a familiar location:

I liked that I decided to take five minutes. And I almost always did it in the morning. And then I did it while lying in bed. So, I was in the same place. [...] I tried it [once] when I was waiting for a bus. But I found that distracting. [...] So that was the good thing about the app. It took five minutes, a routine.

P7; female participant aged 74 y

Some participants enjoyed the fact that the guided mindfulness sessions were short:

Yes, it was very good, actually. And it’s short as well, just five minutes.

P6; male participant aged 71 y

By contrast, others described that they would have enjoyed a longer practice:

I thought they were a bit too short. Five minutes is too short to really get into a relaxed state, or whatever you want to call it. [...] I think ten minutes would make it much better.

P8; female participant aged 83 y

Some participants described challenges in adhering to the mindfulness routines, which was pronounced as a difficulty in finding a suitable time for digital mindfulness practice. One participant mentioned that her habits, such as having breakfast during a certain time of the day, made it difficult to incorporate the app into her morning routines:

It’s difficult [...] I’m someone who has very fixed morning routines. So I wake up, and when I wake up, I have to get up and start breakfast immediately.

P10; female participant aged 78 y

Another participant mentioned that sometimes she forgot to use the app due to being distracted by other activities and routines of daily life:

I must admit I forget it on some days. I try to listen in the mornings, and something always gets in the way. Then I forget it.

P14; female participant aged 74 y

Sometimes, participants felt a sense of obligation to conduct the mindfulness training, and missing the daily mindfulness exercises caused a negative feeling. As a participant mentioned, the app provided flexibility in finding a suitable time for training, but this flexibility sometimes resulted in missing exercises:

When you miss it and think, now I haven’t done it today, but I’ll do it tomorrow. Then you feel like you’re missing out. It has a slightly negative feeling—yes, it does.

P5; female participant aged 66 y

#### Ease of Use and Accessibility

In total, 27% (4/15) of the participants stated that the app was relatively easy to use, and none of them reported any major technical problems with the app:

I didn’t have any problems at all. It was easy to use.

P4; male participant aged 75 y

Even if the app was considered accessible in technical terms, the graphical interface and symbols associated with training were sometimes considered confusing:

It should be clear which exercises you’ve done or not. The star was a bit confusing—I did not know what it meant.

P4; male participant aged 75 y

One participant highlighted that they would have preferred titles for the exercises so that it would have been clear what they were about:

I guess it’s better that each exercise would have a title. It’s not clear what it’s about.

P5; female participant aged 66 y

One participant suggested that mindfulness apps can increase the accessibility to mindfulness training, in comparison to practicing yoga in a studio, which she considered to be too expensive for her budget as a pensioner:

I used to go to medicinal yoga. That was really, really good. So I did two semesters. That’s something I miss, but I also miss the money.

P11; female participant aged 79 y

### Theme 2: Heterogeneity in Older Adults’ Design Preferences for Digital Mindfulness

#### Voice and Sound

Voice interaction and sound in the mindfulness exercises were a significant part of the experience*.* Many participants described how the voice of the mindfulness coach impacted the perceived benefits of training, such as the sense of calmness:

He had a good voice. He spoke clearly, and with a sense of calm.

P10; female participant aged 78 y

If the voice of the mindfulness coach was not pleasant or aligned with participants’ preferences, this could have decreased the user experience.

Overall, participants expressed diverse preferences for voice interaction and a need to adjust the voice according to their individual desires. The voice interaction should represent qualities such as calmness, quietness, and meditative techniques:

I think I would like a quieter voice. A slightly more meditative voice. [...] He speaks in some dialect. He has a very special voice. Maybe that’s why I [like] quite a low voice mode. Not so high, but lower. [...] When you try to calm down, you need help with that. A slightly darker voice, not too fast.

P7; female participant aged 74 y

Some participants reported inconsistencies in the sound interaction. In total, 27% (4/15) of the participants described how sudden pauses in the sound disturbed the mindfulness practice, which showed the significance of voice and sound interaction in the mindfulness experience:

I thought the app’s content was quite good, but the way it was done wasn’t great. The recording made it so that, when the guy is talking, it sounds good, but then there are pauses now and then, and all the sound and ambient noise disappear.

P4; male participant aged 75 y

He makes these pauses. They’re so long, and then you wonder if there’s something wrong with the recording.[...] You get distracted by these pauses. Is this right, or is it wrong? And then you kind of wake up.

P14; female participant aged 74 y

Some participants suggested that they would have preferred music in the background to avoid the sudden pauses and to add to the sense of calm:

I feel that music adds a lot, so if you would have calm music playing in the background, I think it would create a stronger feeling. [...] It’s always so nice when there’s a kind of background sound—it’s very soothing.

P3; male participant aged 66 y

#### Mindfulness Theme, Script, and Coach

Mindfulness script and content provoked different metaphors among participants. A participant highlighted the effectiveness of figurative descriptions, particularly the metaphor of a wave:

I liked the figurative descriptions, like when he said, “feel it like a wave.” I felt supported by the app. I had used breathing techniques during pregnancy, but this was completely different.

P1; female participant aged 65 y

The imagery of a wave aligns with common mindfulness techniques, where emotions and sensations are framed as transient, reducing resistance and promoting acceptance. Metaphorical language can enhance the practice by linking abstract concepts (eg, transience) to physical sensations.

The content provided by the mindfulness app did not always align with participants’ real-life experiences. One participant indicated that the mindfulness practices in the app may downplay the complexity of real-life struggles:

In some exercises, near the end, the person speaking—or this app—seemed to think that big problems could just disappear like that. But it’s not that easy.

P6; male participant aged 71 y

This comment reflected frustration or skepticism, indicating that while mindfulness can be helpful, it may not provide sufficient depth for more profound personal issues. This aligned with critiques that mindfulness, when decontextualized, may not fully support users with complex psychological challenges.

Some participants valued the soft and reassuring tone of the mindfulness coach. This was illustrated by one participant as follows:

He [the mindfulness coach] was nice. His tone was soft as well. He also used those kinds of analogies sometimes. I thought that was nice.

P15; female participant aged 77 y

The positive response to the coach’s tone suggested that a calm and warm delivery might enhance the soothing effects of mindfulness. In contrast, some participants perceived the coach’s persona as too immature for their age group, leading to frustration and disengagement:

The guy [mindfulness coach] sounds too childish. He speaks in a way that is a bit immature. It’s annoying. It’s less authoritative. [...] I was a bit annoyed that his texts can be a little cheesy, a bit childish as well and a bit immature. If you’re 65 plus, it’s not very smart. That could bother me.

P4; male participant aged 75 y

The repeated use of “childish,” “cheesy,” and “immature” suggested that some older adults preferred a more mature, authoritative style in mindfulness guidance. The critique implied that users may expect more intellectually engaging or sophisticated content. A less authoritative tone may reduce credibility and make the guidance feel less impactful or serious.

### Theme 3: Consequences of Digital Mindfulness to the Self-Perceived Well-Being of Older Adults

#### Digital Mindfulness as a Facilitator of Resilience

Many participants mentioned an increased bodily awareness as an outcome of mindfulness training. A participant stated that “Breathing exercises are what make you get in touch with the body” (P7, female participant aged 74 y). Another participant who had no previous experience with mindfulness and reported only a low use of other mHealth apps noticed that “I feel when I walk, that my shoulders are lower, since I started doing it, that I became aware of my posture” (P11; female participant aged 79 y)*.* By using the app, participants could adopt and increase their bodily knowledge and make changes to improve their bodily postures independently.

A decrease in muscle tension was also described as a self-perceived benefit of digital mindfulness. When mindfulness training involves paying attention to body parts, this can result in improved awareness of the tensions or discomforts in the body. Listening to digital mindfulness can help participants to imagine alternative bodily postures, which can result in a sense of improved bodily awareness. One participant described increased relaxation in the shoulders as a self-perceived benefit in using the app:

Some of the exercises include feeling for the legs or the stomach and so on. It’s good for bodily awareness. It is very good and useful. [...] For example, if I have pain somewhere, or you are tense somewhere in particular, I tense my shoulders and I can imagine breathing out into my shoulders to relax the shoulders more easily. It’s the kind of thing you can put into practice when you use these guided meditations.

P4; male participant aged 75 y

Breathing exercises are a central part of mindfulness-based training [22]. To some extent, breathing was considered to be a facilitator and associated with all outcomes of mindfulness training, such as bodily awareness, sleep quality, relaxation, and positive thinking. Participants such as P6, who had no previous mindfulness experience, mentioned an increased awareness of breathing:

Breathing—I knew before how important it is, a few deep breaths. But after this exercise, I’m even more motivated, even more aware.

P6; male participant aged 71 y

Participants such as P2 and P7 noticed that mindfulness training was connected to improvements in the breathing technique. One of them mentioned feeling better after the breathing exercises, which helped her to improve her breathing technique:

Instead of just breathing up here all the time, like I often do. I can say, for that reason, that I feel better.

P2; female participant aged 65 y

A participant who had previous mindfulness experience and reported intermediate well-being scores described breathing exercises as a method to pay attention to incorrect breathing techniques, such as holding one’s breath:

I think the breathing exercises are good. I find it interesting that thoughts affect breathing, and that we breathe too little. We hold our breath a lot. So, I’m always training to breathe with my stomach.

P7; female participant aged 74 y

Relaxation was one of the most frequently reported consequences of practicing digital mindfulness through breathing-related exercises, mentioned by 53% (8/15) of the participants. Participants such as P4, P5, and P6 mentioned a sense of calmness, relaxation, and improved focus on the present moment, followed by digital mindfulness training:

We even tested my pulse, and with this smartwatch I have, my pulse goes down. It feels really good.

P6; male participant aged 71 y

Other participants, such as P10 and P12, mentioned a feeling of joy and a sense of renewed energy associated with relaxation after practicing digital mindfulness:

It’s about relaxing. When you wake up after ten minutes, you feel like you’ve come back. You rest, and your body feels fresher.

P10; female participant aged 78 y

For P5 and P6, this sense of relaxation was considered to have cognitive elements, such as maintaining better control of one’s own thinking and thoughts:

You become calmer because thoughts are focused on breathing [...]. Suddenly, your thoughts don’t stick as much, and they might rotate or move away.

P6; male participant aged 71 y

Digital mindfulness practice provoked positive thinking among participants. One participant who had previous mindfulness experience described how the mindfulness app increased the ability for self-reflection and harmony and also being in control of one’s own emotions:

I don’t see myself as someone who complains a lot, but I do sometimes meet many people who sigh over just about everything. So yes, reflecting can help. I really like this idea of sitting for a while—it’s something that guides you, and you settle into a calmer pace, a nice harmony.

P5; female participant aged 66 y

A participant described the emotional awareness associated with the app use. As she described, this awareness of one’s own thinking can provoke new approaches for self-reflection, which provides possibilities to change the way one thinks about oneself. From this perspective, mindfulness training has similarities with cognitive behavioral therapy [60]. The participant had previous experience with mindfulness and intermediate self-perceived well-being:

You get irritated and frustrated, and you get angry at yourself, you are so frustrated by making a mistake—why did I say this? And then I realise that maybe you can handle it in a different way [...] That maybe I can look at my well-being and my inner self in a different way [...]. I often get stressed by something I have done wrong.

P14; female participant aged 74 y

One participant recognized that increasing awareness of breath can help in letting go of negative thoughts. This reflects cognitive defusion, a core principle in mindfulness and acceptance and commitment therapy [61]. Cognitive defusion refers to the process of detaching from thoughts, seeing them as transient mental events rather than absolute truths:

You become calmer, because thoughts go on breathing and focus. And it was a good method, to let the mind rest on the sensation of the breath. Suddenly, thoughts don’t stick very much, and they might rotate or go away. It felt good, I became a bit more conscious, you could say.

P6; male participant aged 71 y

#### Digital Mindfulness as a Therapeutic Intervention

Several participants recognized the mindfulness training as beneficial for supporting their mental health to alleviate a sense of worry or stress. P6 (male participant aged 71 y), who had no previous experience with digital mindfulness and reported high well-being scores, mentioned mindfulness training as a good method to decrease a sense of worry about “world events, worries about my family—my children, grandchildren.”

During the interviews, P2 (female participant aged 65 y) and P4 (male participant aged 75 y), who both had previous experience with mindfulness, shared that they had experienced panic attacks before in life. For this reason, they had used breathing to help calm down and reported a need to find different methods for relaxation. One of them also described his personal experience with having attention-deficit/hyperactivity disorder, which is often accompanied by high energy and heightened emotions. He mentioned that with voice-guided digital mindfulness, he learned new tools to manage this condition:

I think mindfulness is very helpful for people with ADHD. I believe it’s very, very helpful. That’s one reason why I started [mindfulness training]. With ADHD, you have a lot of energy, and your head spins a lot. Learning to manage that better is a big win.

P4; male participant aged 65 y

Breathing techniques embedded in the app created a sense of bodily relaxation, which was considered to improve sleep quality. Two participants used the breathing exercises as a method of relaxation in the evenings, which resulted in them falling asleep faster. In this way, digital mindfulness could be used as an intervention to improve sleep in a self-guided format:

I have trouble sleeping. I sleep for two hours, then I wake up. But then I get up and sit and listen to it [mindfulness app]. If that doesn’t work, I do crosswords for a while, then listen [to the app] again. Then I go back to bed, and I fall asleep.

P11; female participant aged 79 y

What I liked, and which has helped me quite a bit are these breathing exercises. Because it has sort of helped me to fall asleep faster in the evening than I have done before.

P9; female participant 69 y

In total, 33% (5/15) of the participants reported that the mindfulness training with the app helped them in managing pain and physical discomfort. Although the app did not alleviate the pain, as such, it could help redirect one’s thoughts and “disconnect” from the feeling of pain. A participant described that the mindfulness app helped her to cope with chronic pain caused by rheumatism, which caused physical discomfort in her daily life:

I’ve had PMR [polymyalgia rheumatica] for a year now, and it has been so hard [...], there is a lot of pain sometimes. When you sit and just feel that pain, or lie down and just feel that, then it’s very difficult to get your mind off it. But with the mindfulness app, I disconnect from that. So, then the pain disappears.

P11; female participant 79 y

A participant mentioned that increased bodily awareness through mindfulness training helped them accept the pain or physical limitations caused by an injury. This self-perceived benefit was connected to the relaxation that the app provided:

I am right-handed and the injury is on the right side of the neck vertebrae. [...] Of course, mindfulness helps. Thinking about relaxation, even though there’s an injury, helps you manage it in the right way. You learn to relax, and that helps.

P12; female participant aged 66 y

#### Adverse Effects of Digital Mindfulness

Sometimes participants reported adverse or unexpected negative effects from practicing digital mindfulness. One participant described how practicing digital mindfulness resulted in a sense of discomfort and a sense of physical pain because of not being able to find the right position or location for the training. Her statement also indicated that benefiting from digital mindfulness often requires familiarity with finding locations that are optimal for mindfulness training:

When I sit on a chair, I get back pain, and I don’t know if it’s because I’m sitting still, but I feel more pain in my back. I have to move around more. It’s so rare to sit still in a relaxed way. Now I did get back pain, because of the chair. One should sit on a better chair or lie down, but then you might fall asleep.

P14; female participant aged 74 y

Another adverse effect of digital mindfulness was mild emotional distress, as mentioned by P3 and P1. P3, who had no previous mindfulness experience, articulated that mindfulness scripts from the app highlighted unpleasant situations in everyday life that provoked distracting emotions and disturbed his sense of calmness:

It almost became annoying then when he brought up different everyday situations. “Why did I say that? Why did I do that?” I don’t want to think about that; I just want to continue with the calmness.

P3; male participant aged 66 y

Similarly, one of the participants described how breathing exercises generated unpleasant memories from a personal life event. For her, this emotional distress was associated with the breathing activity:

Breathing is emotionally charged for me because my brother passed away from ALS [Amyotrophic Lateral Sclerosis], which makes it hard to breathe. So it made me think about that. I’ve had quite a bit of pain at times in my life—not really now—but it also reminded me of that.

P1; female participant 65 y

## Discussion

### Principal Findings

#### Overview

Previous studies have explored the effects of mindfulness training on the well-being of older adults through randomized controlled trials from the medical or clinical perspective [16-18]. The research evidence indicates the positive effects of mindfulness training on physical well-being [26,27]. Previous studies have also confirmed that MBIs can reduce negative emotions and thoughts in daily life among the general population [24,25,27] and demonstrated the effectiveness of MBIs to reduce stress, anxiety, and depression among older adults [4,17,28,29,62,63]. However, the research evidence on the specific characteristics of voice-guided digital mindfulness for mental and emotional well-being among older adults is rare. This study has filled this research gap by exploring voice-guided digital mindfulness and emphasizing the subjective experiences of digital mindfulness and their possible effects and outcomes among older adults.

#### Creating Routines With Digital Mindfulness Through Temporalities

As a response to the first research objective, the study has characterized the specific conditions for embedding the mindfulness app into the daily routines of older adults, which can be summarized as mentioned subsequently.

First, in order to actively benefit from digital mindfulness, older adults need to integrate the use of the app into their daily routines and schedules. In general, participants found the digital mindfulness app easy to incorporate into their daily schedules, with the flexibility to choose sessions at times that suited their personal routines. The brevity of the sessions, typically approximately 5 minutes, was appreciated by many participants, facilitating regular practice without significant time commitment. This highlights how the flexibility of digital platforms allows personalized engagement, enabling users to select practices that align with their individual preferences and schedules.

Second, interestingly, the challenges in the adherence to mindfulness training were not associated with technical difficulties or the accessibility of the app but rather with the extent to which older adults could find a suitable time to practice digital mindfulness. This underscores the need for apps to provide reminders or integrate seamlessly with users’ existing schedules to promote adherence and aligns with existing research highlighting that continuity in app use can be a challenge [40].

Third, although the app itself enabled practicing digital mindfulness “anywhere and anytime,” older adults often chose the location and time based on their existing daily habits, thus embedding the app into existing routines and practices. Participants highlighted how the effects of mindfulness varied according to what time of the day they practiced.

#### Heterogeneous Design Preferences for Voice-Guided Mindfulness

As a response to the second research objective, this study has recognized heterogeneity in the design preferences for voice-guided digital mindfulness. Practicing digital mindfulness is a highly personalized experience, influenced by a person’s previous mindfulness experience, health conditions, and motivations for training [32,64]. This study shows that this heterogeneity is particularly pronounced in relation to design preferences of digital mindfulness apps: voice and sound landscape and mindfulness theme, script, and coach persona. The diversity in older adults’ experiences and preferences with digital mindfulness underscores the necessity for customizable voice-guided apps. This variability suggests that a one-size-fits-all approach may not be effective, and developers should consider offering adjustable features, such as voice tone, session length, and content complexity, to cater to the unique needs of each user.

This study indicates that design preferences can partly be associated with various emotions and sensations that participants associate with certain types of voices, sounds, and scripts. For instance, the description of some mindfulness scripts as too “childish” or “immature” for older adults is a sign that mindfulness content should be adapted to age-specific characteristics. While other studies have revealed preferences for personalization of a mindfulness app among the younger population [65], the age-specific characteristics for certain interaction modalities or conversational styles have not been explored. This study concludes that the audio landscape of mindfulness apps provides possibilities for enhancing the mindfulness experience in a way that can respond to the age-specific characteristics of this age group.

#### Building Resilience and Self-Care Through Digital Mindfulness

As a response to the third research objective, this study has addressed older adults’ self-perceived experiences of using digital mindfulness as a self-care technology to facilitate well-being. Previous research shows that mindfulness is useful for improving resilience and coping strategies and reducing anxiety and stress levels in older adults [62,63,66]. This study highlighted that digital mindfulness practices could contribute to resilience by fostering positive thinking, bodily awareness, relaxation, and self-reflection. Participants noted a greater ability to manage negative emotions and adopt new perspectives on personal well-being. Beyond emotional benefits, participants perceived digital mindfulness to be a therapeutic tool for managing mental health concerns and pain and improving sleep quality. Participants reported that mindfulness practices helped redirect focus away from pain, providing a sense of relief. This aligns with studies suggesting that web-based mindfulness interventions can lead to improvements in cognitive and psychological measures, with associated modulations in pain perception.

However, the adverse effects of digital mindfulness were also pronounced, in terms of discomfort and emotional distress that resemble re-experiencing unpleasant life events [32]. These findings underscore the importance of personalized mindfulness interventions and the potential need for guidance on adapting mindfulness practices to individual emotional and physical comfort levels.

By incorporating mindfulness practices into their routines, older adults can develop better coping mechanisms to navigate stressors and challenges. The convenience of digital platforms ensures that these resources are readily available, promoting sustained engagement and long-term benefits.

### Limitations

This study has explored the use of a mindfulness app as a form of digital self-care among older adults through the lens of aged heterogeneity. Participants were recruited through a municipal organization, which means that research participants were relatively active older adults with a previous interest in health and well-being. Moreover, participants used the app for 3 weeks; therefore, the long-term effects of mindfulness app use are not known. This study focused on the self-perceived benefits of digital mindfulness, as articulated by participants themselves, which means that the actual use patterns remained unexplored in this study. The use, design preferences, and consequences to well-being of the mindfulness app could depend on contextual factors, such as mood, digital skills, and education level, which remain beyond the scope of this study. Despite these limitations, this study has produced new knowledge on the self-perceived benefits of digital mindfulness among older adults, particularly regarding the affective aspects of mindfulness app use and the integration of the app into daily routines. These qualitative findings extend the already existing medical and clinical research on digital mindfulness with older adults [14-16,18] and provide considerations for designing MBIs beyond medical or clinical purposes, such as in the maintenance of cognitive or physical functioning in later life.

### Conclusions

The study concludes that digital mindfulness offers a possibility to enhance the self-perceived well-being of older adults by fostering resilience and self-care. However, the adverse effects of digital mindfulness, such as frustration and discomfort, can also be experienced by older adults. The digital mindfulness experiences and preferences of older adults are highly individual, diverse, and manifold, which indicates that personalized approaches are essential for effective engagement. By acknowledging and addressing the heterogeneous design preferences within this demographic, developers can create more effective and user-friendly voice-guided mindfulness apps. Additional research is needed to explore the emerging digital inequalities involving older adults from a more diverse socioeconomic background to explore the benefits of digital mindfulness in relation to an individual’s digital health literacy. Furthermore, longitudinal studies could provide deeper insights into the sustained effects of digital mindfulness on well-being over time. Future research should also explore ways to personalize digital mindfulness experiences, considering factors such as previous mindfulness experience, individual emotional triggers, and physical limitations. Future studies should also explore the potential of artificial intelligence to enhance the personalization of mindfulness experience to be more adaptive to older adults’ previous mindfulness experience and their personal preferences for certain types of voices, sounds, and mindfulness coaches’ personas.

## Appendix

Multimedia Appendix 1 Interview topic guide.

Multimedia Appendix 2 Thematic analysis of interview data.

## Abbreviations

FFMQ: Five Facet Mindfulness Questionnaire
MBI: mindfulness-based intervention
MBSR: mindfulness-based stress reduction
mHealth: mobile health
WHO-5: World Health Organization-Five Well-Being Index

## Data Availability

The datasets generated or analyzed during this study are available from the corresponding author on reasonable request.
