# Supplementary material for: Design Preferences, Routines, and Well-Being of Older Adults Using Voice-Guided Digital Mindfulness: Qualitative Interview Study
Source: JMIR Hum Factors. 2025 Jun 3;12:e67533. doi: 10.2196/67533 (PMC12174884; doi:10.2196/67533)
Supplement: Multimedia Appendix 1 [file humanfactors_v12i1e67533_app1.docx]

**Multimedia Appendix 1**. Interview topic guide (Translated from Swedish)

[V1; 1 May 2024]

This is a semi-structured interview, and thus the questions below provide a guide – not a script – in conducting the interviews. Some questions below may be omitted, and others added, depending on where the participant takes the conversation.

**Introduction:**

- Ensure the participant is in a comfortable location where they are happy to talk
- Briefly introduce the project again and the purpose of the interview
- Obtain verbal consent to participate and record the conversation
- Introduce myself and my experience with mindfulness again

**Perceived health:**

- How do you perceive your physical health at present?
- How do you perceive your mental health at present?
- Do you have any health problems that could affect your ability for practising mindfulness?
- Do you have any other conditions in your life that could affect your motivation for mindfulness training?

**Routines:**

- Did you feel motivated to use the app every day? Why/why not?
- How often did you use the app?
- At what time of day did you use the application? Why?
- Has the app changed your daily routines in any way?
- Do you think you will continue to use the app? Why/why not?

**Design preferences and usability:**

- Did you think the app was easy to use?
- Did you experience any difficulties when using the app?
- Do you think you would need help to download or know how to use the app?
- Did you find the app unnecessarily complicated?
- Did you find any of the information in the app useful?
- What did you think about the feedback from the app? Would you have liked to have more?
- Did you like the voice of the mindfulness coach? Did you like the personality? Why/why not?

**Digital mindfulness and well-being:**

- Did you feel that practising digital mindfulness affected your well-being? In what way?
- Has using the app affected your health in any way?
- Did you experience any positive feelings when you used the application? Which ones?
- Did you experience any negative feelings when you used the application? Which ones?
- Did you experience any positive physical sensations when you used the application? Which ones?
- Did you experience any negative physical sensations when you used the application? Which ones?

**Ethical considerations:**

- Did you feel that the guidance in the app was trustworthy?
- Did the app ever cause you distress, discomfort, or anxiety? If so, in what way?
- Were there any aspects of the mindfulness exercises that felt inappropriate or culturally insensitive to you?

**Open question before debriefing**: is there anything else about your experience of using the mindfulness application that you have not had the chance to share yet?

**Debrief:**

- Check in with how the participant feels after the interview
- Thank the participant for their feedback and participation
- Offer email for follow-up contact and for a copy of report when finished
